# Colorectal Cancer Cell-Derived Extracellular Vesicles Promote Angiogenesis Through JAK/STAT3/VEGFA Signaling

**DOI:** 10.3390/biology13110873

**Published:** 2024-10-27

**Authors:** Yuqing Long, Yuxi Dan, Yao Jiang, Jing Ma, Tao Zhou, Liaoqiong Fang, Zhibiao Wang

**Affiliations:** 1 State Key Laboratory of Ultrasound in Medicine and Engineering, College of Biomedical Engineering, Chongqing Medical University, Chongqing 400016, China; aoko@stu.cqmu.edu.cn (Y.L.); cqmudyx@stu.cqmu.edu.cn (Y.D.); jyao@stu.cqmu.edu.cn (Y.J.); cqmumj@stu.cqmu.edu.cn (J.M.); zhoutao2@stu.cqmu.edu.cn (T.Z.); 2 Chongqing Key Laboratory of Biomedical Engineering, Chongqing Medical University, Chongqing 400016, China; 3 National Engineering Research Center of Ultrasound Medicine, Chongqing 401121, China

**Keywords:** extracellular vesicles, angiogenesis, colorectal cancer, VEGFA, JAK/STAT3

## Abstract

Background: Angiogenesis plays a crucial role in the growth of colorectal cancer (CRC). Recent studies have identified extracellular vesicles (EVs) in the tumor microenvironment as important mediators of cell-to-cell communication. However, the specific role and mechanisms of CRC-derived EVs in regulating tumor angiogenesis remain to be further investigated. Methods: EVs were isolated from the conditioned medium of the CRC cells using ultracentrifugation. We investigated the effects of HT-29-derived EVs on tumor growth and angiogenesis in a subcutaneous HT-29 CRC tumor model in mice. Additionally, we evaluated the impact of HT-29-derived EVs on the proliferation, migration, and tube formation of human umbilical vein endothelial cells (HUVECs). Subsequently, bioinformatics analysis was performed to identify relevant signaling pathways, and pathway inhibitors were used to block the activation of these pathways, aiming to elucidate their roles in angiogenesis. Results: We found that HT-29-derived EVs can promote tumor growth and angiogenesis in vivo, as well as significantly enhance the proliferation, migration, and tube formation of HUVECs. Bioinformatics analysis revealed that HT-29-derived EVs may regulate angiogenesis through the JAK/STAT3 signaling pathway. Specifically, we observed that CRC-derived EVs promoted the phosphorylation of STAT3 (p-STAT3) and the expression of VEGFA in the nucleus of HUVECs. Treatment with the STAT3 inhibitor Stattic reduced the nuclear expression of p-STAT3, which impaired its function as a transcription factor, thereby inhibiting VEGFA expression and the pro-angiogenic effects of CRC-derived EVs. Conclusions: EVs derived from CRC cells promote CRC tumor angiogenesis by regulating VEGFA through the JAK/STAT3 pathway in endothelial cells.

## Simple Summary

Colorectal cancer growth and metastasis depend on blood vessels to supply the tumor with essential nutrients and oxygen. Tumor cells release angiogenic signals to support their progression. While tumor-derived extracellular vesicles (EVs) are recognized for their role in communication within the tumor microenvironment, the specific mechanisms by which they regulate angiogenesis require further exploration. Our study found that extracellular vesicles derived from CRC cells can promote tumor growth and angiogenesis in a mouse model. Cell experiments revealed that endothelial cells can uptake these EVs, leading to enhanced proliferation, migration, and tube formation. Through bioinformatics analysis and experimental validation, we discovered that CRC-derived EVs regulate angiogenesis by modulating the JAK/STAT3 signaling pathway in endothelial cells, resulting in increased VEGFA expression and promoting angiogenesis.

## 1. Introduction

Tumor angiogenesis is vital for the growth of tumors as it provides the essential nutrients and oxygen necessary for tumors [1]. To grow beyond a size of approximately 1–2 mm^3^, a tumor requires the establishment of new blood vessels. During this period, tumor cells release increased proangiogenic signals, disrupting the stability of blood vessels and promoting the formation of new blood vessels [2]. Colorectal cancer (CRC) is the third most common malignancy and the second most deadly cancer [3]. Angiogenesis is also a critical process in its progression [4]. In fact, as early as 1971, Judah Folkman proposed that tumor growth relies on angiogenesis and suggested that inhibiting angiogenesis is a promising strategy for cancer treatment [5]. However, due to the still incomplete understanding of the mechanisms of tumor angiogenesis, the success of this strategy remains limited. Therefore, it is essential to study the potential proangiogenic signals involved in tumor angiogenesis, which may provide an effective approach to the treatment of cancer.

Extracellular vesicles (EVs) are nano-sized, membranous structures secreted into the extracellular space [6]. They contain a variety of functional biomolecules, including proteins, RNA, and DNA [7]. Research shows that EVs can mediate cell-to-cell communication via transporting bioactive molecules from donor cells to recipient cells [8]. Tumor cells release EVs into the tumor microenvironment (TME), mediating communication between tumor cells and the surrounding cells and thereby promoting cancer progression [9]. Endothelial cells are the most crucial effector cells in angiogenesis [10]. Tumor cells can influence the biological behavior of endothelial cells through cell communication mediated by EVs, thereby affecting tumor angiogenesis [11]. Therefore, investigating the mechanisms by which tumor-derived EVs promote angiogenesis in endothelial cells may provide valuable potential therapeutic targets for the treatment of cancer. However, further research is needed to understand how EVs derived from CRC regulate the mechanisms of tumor angiogenesis.

In this study, we demonstrated that extracellular vesicles derived from HT-29 colorectal cancer cells (HT29-EVs) promote HT-29 colorectal cancer tumor growth and angiogenesis in mice. Subsequent investigations revealed that HT29-EVs can be internalized by human umbilical vein endothelial cells (HUVECs), enhancing their proliferation, migration, and tube formation. Our further investigations have revealed that CRC-EVs promote angiogenesis by regulating the JAK/STAT3 pathway in HUVECs. This study establishes the role of CRC-EVs in promoting CRC tumor angiogenesis and elucidates the molecular pathway involved. Therefore, our work has the potential to enhance our understanding of tumor angiogenesis in CRC and further elucidate the role of tumor-derived EVs in tumor development. It provides a theoretical basis for targeting EVs in anti-tumor therapy for CRC, as well as for the prevention and treatment of other tumors.

## 2. Materials and Methods

### 2.1. Cell Lines and Cell Culture

Human Umbilical Vein Endothelial Cells (HUVECs), the human colorectal cancer cell lines SW480, and HCT116 were obtained from the American Type Culture Collection (ATCC, Manassas, VA, USA). The human colorectal cancer cell line HT-29 was obtained from Shanghai Zhong Qiao Xin Zhou Biotechnology Co., Ltd. (Shanghai, China). HUVECs, HT-29 and SW480 were maintained in Dulbecco’s Modified Eagle Medium (DMEM) (GIBICO, Waltham, MA, USA) supplemented with 10% fetal bovine serum (FBS). HCT116 was maintained in McCoy’s 5A Medium (GIBICO, Waltham, MA, USA) supplemented with 10% FBS.

### 2.2. Isolation and Characterization of Extracellular Vesicles

Extracellular vesicles were purified from the conditioned medium of HT-29, SW480, and HCT116 cells using ultracentrifugation. We used an ultrafiltration-based protocol to deplete extracellular vesicles from FBS [12]. When the cells reached 80% confluency, the supernatant was collected. The collected supernatant was centrifuged at 800× *g* for 5 min at 4 °C to remove cells, followed by centrifugation at 2000× *g* for 10 min to remove cell debris and centrifugation at 10,000× *g* for 30 min at 4 °C to remove large vesicles. To collect more extracellular vesicles per ultracentrifugation, we used a 100 kDa centrifugal filter (Merck Millipore, Darmstadt, Germany) to concentrate EVs and filter out small proteins [13]. The supernatant was centrifuged at 100,000× *g* for 90 min at 4 °C, resuspended with PBS, and then centrifuged under the same conditions. The obtained particles were resuspended in PBS. The number of EVs was measured using the bicinchoninic acid (BCA) protein assay kit (Beyotime Biotechnology, Shanghai, China). EVs were stored at −80 °C and thawed before use.

The characterization of the EVs was verified by detecting the expression of the EV-positive markers CD63 (1:2000; ab134045, Abcam, Cambridge, UK) and TSG101 (1:2000; ab133586, Abcam, Cambridge, UK), as well as the negative marker calnexin (1:500; Abcam, Cambridge, UK), using Western blotting analysis. The morphology of EVs was observed with a transmission electron microscope (TEM; FEI TECNAI G2, Hillsboro, OR, USA). EVs were fixed with 1% glutaraldehyde in PBS and then spotted onto 300-mesh carbon/formvar-coated grids. The grids were stained for contrast using uranyl acetate in water for 10 min and imaged with TEM. The particle size of EVs was measured using NanoSight NS300 (Malvern, UK).

### 2.3. Uptake of HT29-EVs

After coincubating EVs and PKH67 (Solarbio, Beijing, China) for 30 min, EVs were labeled with fluorescent PKH26. Subsequently, the PKH26-labeled EVs were washed in PBS and centrifuged at 100,000× *g* for 90 min at 4 °C to collect EVs, which were then resuspended in PBS for further analysis. After incubating PKH67-labeled EVs with HUVECs for 6 h, we stained the nuclei of the HUVECs using DAPI and viewed them under a fluorescent microscope (Andor Dragonfly, London, UK).

### 2.4. Proliferation, Migration, and Tube Formation Assays

HUVECs proliferation was detected using an EdU assay after 24 h of culture with EVs. A fluorescent microscope was used to observe the samples and obtain images.

Cell migration was analyzed using a transwell and scratch wound healing assay. The transwell assay was described previously [14]. In brief, 200 μL of serum-free DMEM containing HUVECs (1 × 10^4^ cells/well) was added to each upper transwell chamber (8 μm pore size; Corning, New York, NY, USA) and 600 μL of DMEM (10% FBS) with different concentrations of EVs was poured into each lower chamber. The plate was incubated in an incubator for 24 h. The cells on the filter membrane of the transwell insert were carefully removed. Then, we fixed them with paraformaldehyde for 15 min and washed them three times with PBS. Crystal violet staining was performed, and the number of penetrated cells was determined in five randomly selected high-power fields for each membrane. For the scratch wound healing assay, HUVECs were seeded into 6-well tissue culture plates at a density of 4 × 10^5^ cells/well. After 24 h, a scratch was made in the monolayer with a sterile 10 μL pipette tip and washed with PBS. Subsequently, 2 mL of serum-free media containing varying concentrations of EVs was added. The wound was imaged with a microscope at a 4× magnification after 0 and 24 h. The wound area was analyzed using ImageJ software (https://imagej.net/software/imagej/, accessed on 24 October 2024). The percentage of wound closure was calculated.

For the tube formation assay, HUVECs at 2 × 10^4^ cells/well were cultured in 96-well plates that had been coated with Matrigel (50 μL/well; Beyotime Biotechnology, Shanghai, China) for 6 h and imaged with a microscope. ImageJ software was used to analyze the number of nodes, total tube length, and total loops. In the experiment investigating the effects of HT29-EVs at different concentrations on HUVECs, the HT29-EVs protein concentrations used were 2.5 μg/mL, 5 μg/mL, and 10 μg/mL, with PBS as the control.

In the inhibition experiment, Stattic at a concentration of 1.25 μM (HY-13818, Med-ChemExpress, Shanghai, China) and CRC-EVs at a protein concentration of 10 μg/mL were used. Since the solvent for Stattic is DMSO, considering the toxicity of DMSO to cells, we added an equal volume of DMSO as a control to exclude the influence of DMSO on the cells.

### 2.5. Animal Studies

Male BALB/c nude mice (6–8 weeks) were purchased from Hunan SJA Laboratory Animal Co., Ltd. (Changsha, China) and allowed to acclimate for one week before use. All mice were maintained in a pathogen-free animal facility with a 12-h light/dark cycle. HT-29 cells (1 × 10^7^ cells/mouse) were injected subcutaneously into the flank of nude mice. When tumors became palpable (9 days), mice were randomly divided into two groups (five mice/group) and received intratumoral injections of EVs (EVs protein concentration of 0.25 mg/kg) or PBS every other day for tumor treatment. Tumor volume (V) was measured with calipers every other day and calculated using the standard formula: V = length × width^2^/2. Mice were euthanized on the 20th day of the tumor cell injection, and the tumor tissues were isolated and weighed.

### 2.6. Immunohistochemistry

Immunohistochemistry (IHC) was described previously [15]. The microvessel density (MVD) and levels of VEGFA expression in tumor tissue sections were characterized by IHC using anti-CD31 (1:100; 77699, CST) or anti-VEGFA (1:250; 19003-1-AP, Proteintech, Wuhan, China). MVD was quantified based on CD31-positive endothelial cells in tumor samples assayed by IHC. MVD is a surrogate marker that expressly reflects tumor angiogenesis [16]. The method of quantifying the MVD was described previously [17].

### 2.7. Western Blotting Analysis

Proteins were processed with gel electrophoresis and transferred onto membranes, which were blocked with 5% BSA and incubated with the primary antibodies VEGFA (1:1000; ab214424, Abcam), STAT3 (1:2000; 4904, CST), p-STAT3 (1:2000; 9145, CST), and GAPDH (1:1000; Abcam) and with a relative secondary antibody. After development with an enhanced chemiluminescent reagent, bands were exposed on the Azure Imager C300 System (Azure Biosystems, Dublin, CA, USA). A stripping buffer (Solarbio, Beijing Solarbio Science & Technology Co., Ltd., Beijing, China) was used to elute the antibodies on the PVDF membrane.

### 2.8. Immunofluorescence

Immunofluorescence (IF), as described previously [18], was performed using an anti-PCNA antibody (1:100; AF1363, Beyotime Biotechnology, Shanghai, China), an anti-VEGFA antibody (1:200; 19003-1-AP, Proteintech, Wuhan, China) and anti-p-STAT3 antibody (1:200; 9145, CST, Boston, MA, USA). In brief, after fixation in 4% paraformaldehyde, the cells were rinsed in PBS buffer containing 0.05% Tween-20 prior to Triton X-100-mediated permeabilization. Subsequently, the cells were blocked with 1% BSA for 1 h and incubated overnight at 4 °C with the primary antibody anti-VEGFA. followed by rinsing and incubation for 1 h with the secondary antibody Goat anti-Rabbit IgG/FITC (1:200; SF134, Solarbio, Beijing, China). They were then incubated for 1 h with a secondary antibody Goat anti-Rabbit IgG/FITC (1:200; SF134, Solarbio), and then rinsed and stained with DAPI. The amounts of each protein were quantified using the ImageJ Software.

### 2.9. F-Actin Cytoskeleton Staining Assay

The FITC-Phalloidin staining assay was conducted to investigate the effect of EVs on F-actin in HUVECs. HUVECs were co-cultured with EVs for 24 h. The cells were washed three times with PBS and then fixed in 4% paraformaldehyde at room temperature for 15 min. After fixation, the cells were rinsed in PBS buffer containing 0.05% Tween-20 before permeabilization with Triton X-100. Then, the cells were stained with rhodamine-phalloidin for 20 min. Finally, the nuclei were stained with DAPI.

### 2.10. Bioinformatics Analysis

The microRNA profiles (GSE40247) of normal human fetal colon-derived FHC cells and HT-29 cells EVs were obtained from Gene Expression Omnibus (https://www.ncbi.nlm.nih.gov/geo/, accessed on 25 June 2023), a free and publicly available database [19]. In this study, differential expression miRNAs (DEMs) between HT-29-derived EVs and FHC-derived EVs (FHC-EVs) were screened using the GEO2R online tool (https://www.ncbi.nlm.nih.gov/geo/geo2r/, accessed on 25 June 2023). miRNAs within the cutoff criteria of a *p*-value < 0.05 and |log2 (Fold change)| ≥ 2 were designated DEMs. The experimentally validated microRNA target interaction database, miRTarBase (https://mirtarbase.cuhk.edu.cn/~miRTarBase/miRTarBase_2019/php/index.php, accessed on 25 June 2023), was used to predict the potential target genes of DEMs. The interaction among downstream target proteins was analyzed using the STRING website, and the network was visualized using Cytoscape software (version 3.8.2). CytoHubba, a Cytoscape plugin, was utilized to explore PPI network hub genes [20]. The GO annotation and KEGG pathway enrichment analysis were performed using the R package “clusterProfiler” [21]. This work considered only the terms/pathways with a false discovery rate (FDR) < 0.05, indicating significant enrichment.

### 2.11. Statistical Analysis

Each experiment was repeated at least three times. Statistical analyses were carried out using GraphPad Prism 9.5.0 (GraphPad Software, Boston, MA, USA). Differences between the groups were analyzed using Student’s *t*-test (unpaired, two-tailed) or one-way ANOVA. Data are presented as the mean ± SD. * *p* < 0.05, ** *p* < 0.01, and *** *p* < 0.001 were considered significant differences.

## 3. Results

### 3.1. Isolation and Characterization of HT29-EVs

**Figure 1:**
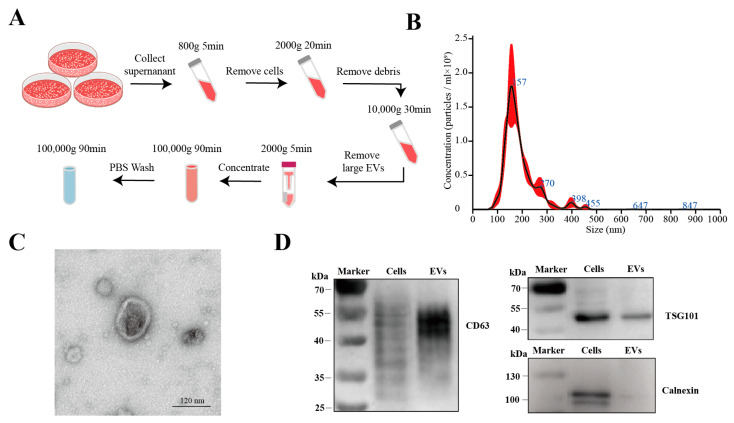
Isolation and characterization of EVs derived from HT-29 colorectal cancer cells (HT29-EVs). (**A**) Flowchart of the extracellular vesicles isolation procedure. (**B**) Nanoparticle tracking analysis of the size distribution of the extracellular vesicles (Figure S9). (**C**) Transmission electron microscopy image of the extracellular vesicles (Figure S10). (**D**) Western blotting analysis of the positive (CD63 and TSG101) and negative (calnexin) markers of extracellular vesicles (Figure S11).

We successfully isolated extracellular vesicles from the HT-29 cell culture supernatant using differential and ultracentrifugation techniques (Figure 1A). Nanoparticle tracking analysis (NTA) indicated that the size distribution of the EVs was mainly in the range of 100–300 nm, which aligns with the sizes reported in the literature (Figure 1B). Transmission electron microscopy (TEM) revealed that the EVs exhibited cup-shaped, round, or elliptical lipid bilayer vesicular structures (Figure 1C). Moreover, Western blotting was performed to determine the presence or absence of EVs’ positive (CD63 and TSG101) and negative (calnexin) markers (Figure 1D).

### 3.2. HT29-EVs Promote the Growth and Angiogenesis of HT-29 Colorectal Cancer Tumors In Vivo

**Figure 2:**
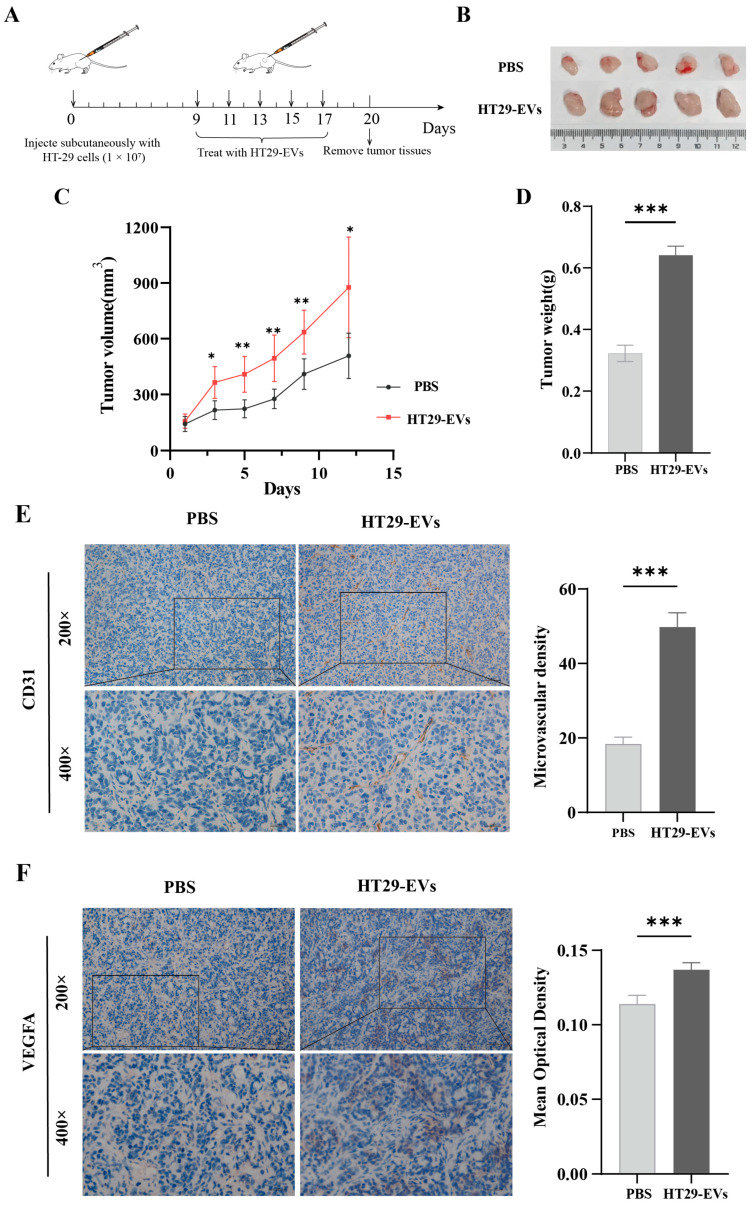
HT-29-derived EVs promote HT-29 colorectal cancer tumor growth and angiogenesis in vivo. (**A**) Model diagram of mouse tumorigenesis model. (**B**) Tumor image of each group. (**C**) The tumor growth curve shows the tumor size measured every 2 days. (**D**) The weight of tumors in each group. (**E**) CD31 staining of tumors and quantification of vessel area (200×: scale bar = 100 μm, 400×: scale bar = 50 μm). (**F**) VEGFA staining of tumors and quantification of the average optical density (200×: scale bar = 100 μm, 400×: scale bar = 50 μm). Data were analyzed by *t*-test, * *p* < 0.05, ** *p* < 0.01, *** *p* < 0.001.

To investigate whether HT29-EVs induce HT-29 colorectal cancer angiogenesis, HT-29 cells were subcutaneously injected into nude mice, and then HT29-EVs were injected into the tumor every 2 days, as shown in Figure 2A. We found that compared to the PBS control group, the tumor volume and tumor weight in mice treated with HT29-EVs were significantly increased (Figure 2B–D). IHC staining showed that HT29-EVs were able to promote CD31 expression in tumor tissues, indicating a higher MVD compared with the PBS control group (Figure 2E). In addition, IHC staining showed increased VEGFA expression after HT29-EVs treatment (Figure 2F). In conclusion, these results indicate that EVs from HT-29 cells promote tumor growth and angiogenesis in HT-29 colorectal cancer tumors.

### 3.3. HT29-EVs Are Taken Up by HUVECs and Promote Their Proliferation, Migration, and Tube Formation

**Figure 3:**
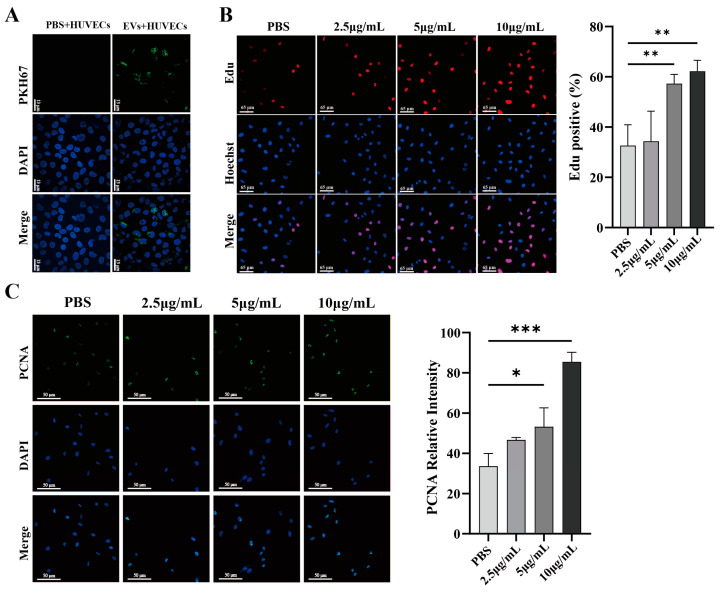

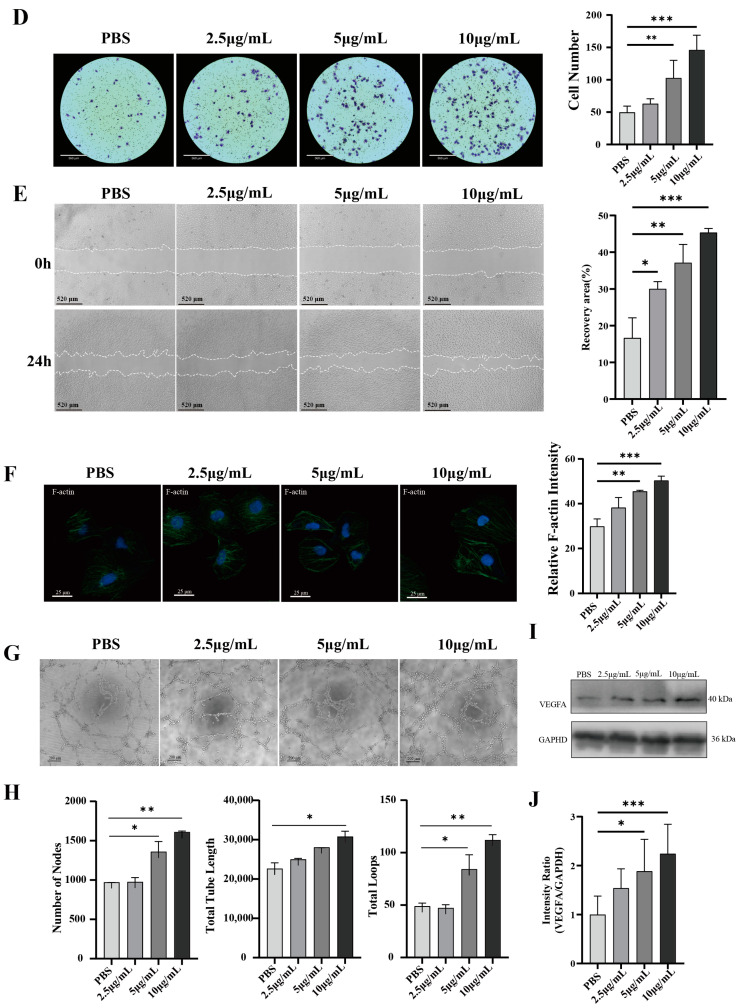
HT29-EVs promote angiogenesis in vitro. (**A**) HUVECs were treated with control PBS or HT29-EVs that had been labeled with PKH67 (green) for 6 h and stained with DAPI (blue) (scale bar = 13 μm). HT29-EVs uptake by HUVECs was observed. (**B**) The EdU assay indicated the effect of different concentrations of HT29-EVs on the proliferation rate of HUVECs (scale bar = 65 μm). (**C**) Immunofluorescence analysis of PCNA protein expression in HUVECs treated with different concentrations of HT29-EVs and fluorescence intensity statistics (scale bar = 25 μm). (**D**) The transwell assay was performed to detect the impact of different concentrations of HT29-EVs on the migration of HUVECs. (**E**) The scratch wound healing assay was performed to detect the impact of different concentrations of HT29-EVs on the migration of HUVECs (scale bar = 520 μm). (**F**) The FITC-phalloidin assay was performed to detect the effect of different concentrations of HT29-EVs on F-actin protein expression in HUVECs (scale bar = 25 μm). (**G**) The tube formation assay was used to detect the angiogenesis ability of HUVECs (scale bar = 200 μm). (**H**) Quantitative analysis of nodes, total tube length, and total loops (Figure S12). (**I**) Western blotting analysis of VEGFA in HUVECs incubated with different concentrations of HT29-EVs. (**J**) Quantitative analysis of VEGFA expression in HUVECs treated with different concentrations of HT29-EVs. Data were analyzed by one-way ANOVA, * *p* < 0.05, ** *p* < 0.01, *** *p* < 0.001.

To determine whether HUVECs uptake HT29-EVs, we co-cultured HT29-EVs with HUVECs for 6 h. Confocal microscopy showed that PKH67-labeled EVs surrounded the cell nuclei labeled with DAPI, indicating the uptake of HT-29 cell-derived EVs by HUVECs (Figure 3A). To investigate the impact of HT29-EVs on HUVECs, a series of experiments, including an EdU cell proliferation assay, a transwell assay, and a tube formation assay, were performed, along with immunofluorescence observation of proliferating cell nuclear antigen (PCNA) protein and cytoskeleton F-actin expression. EdU cell proliferation assays revealed that treatment with HT29-EVs for 24 h significantly increased the proliferation of HUVECs compared with PBS (Figure 3B), and the expression of PCNA was also significantly increased (Figure 3C). Additionally, treatment with HT29-EVs for 24 h significantly promoted HUVECs migration (Figure 3D,E). The cytoskeleton F-actin plays an important role in cell migration [22]. Our study also found that treatment with HT29-EVs increased cytoskeleton F-actin protein expression and filament arrangement of HUVECs (Figure 3F). Moreover, HT29-EVs dramatically promoted the tube formation capacity of HUVECs and increased the number of nodes, total tube length, and total loops in HUVECs tubular networks (Figure 3G,H). In tumor angiogenesis, the vascular endothelial growth factor (VEGF) and its receptors are considered the most crucial factors, and VEGFA occupies the leading role [23]. Therefore, we examined the expression of VEGFA and observed that HT29-EVs enhanced the expression of VEGFA (Figure 3I,J). Together, these data demonstrate that HT29-EVs play a positive role in angiogenesis in vitro.

### 3.4. HT29-EVs Regulate Angiogenesis Possibly Through the JAK/STAT3 Pathway

**Figure 4:**
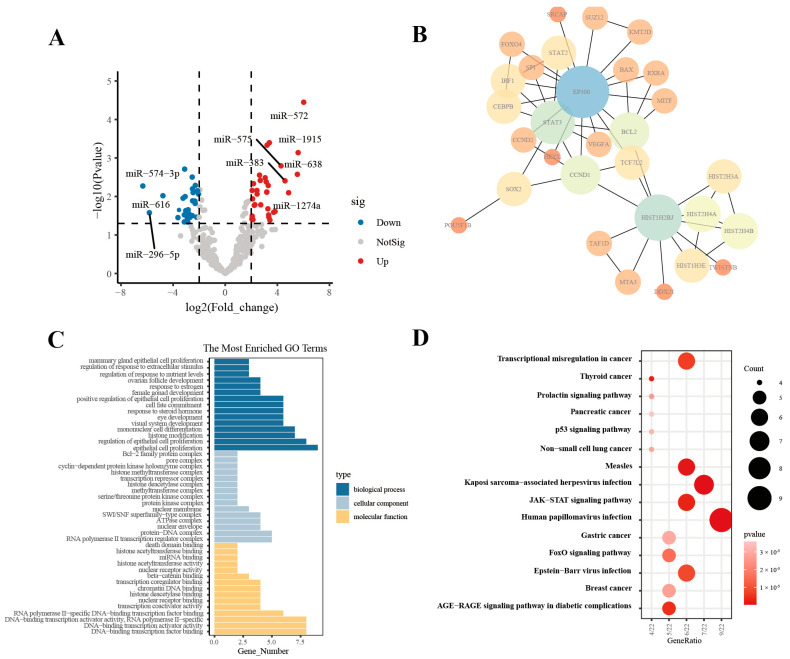
HT29-EVs regulate angiogenesis possibly through the JAK/STAT3 pathway. (**A**) Differential expression profile of miRNAs in EVs derived from HT-29 and FHC cell lines from the GSE40247 dataset. (**B**) The top 30 hub genes for the target genes of DEMs between HT29-EVs and FHC-EVs. (**C**) GO enrichment analysis of the hub genes of target genes for DEMs between HT29-EVs and FHC-EVs. (**D**) KEGG pathway analysis of the hub genes of target genes for DEMs between HT29-EVs and FHC-EVs.

In order to explore the potential mechanisms of HT29-EVs to promote angiogenesis, we analyzed the miRNA sequencing data of EVs derived from HT-29 and normal human fetal colon-derived FHC cells (FHC-EVs) in the GSE40247 dataset [24]. Firstly, we performed differential expression analysis of the miRNA profiles of two types of EVs to identify miRNAs that may play a functional role in HT29-EVs. We found that compared with FHC-EVs, there are 48 upregulated and 43 downregulated miRNAs in HT29-EVs (Figure 4A). We performed target gene prediction for the eight most significantly differentially expressed miRNAs (DEMs) in HT29-EVs, including miR-574-3p, miR-296-5p, miR-383, miR-572, miR-616*, miR-638, miR-1274a, and miR-1915, and a total of 788 target genes were predicted. To explore the interactions among target genes, a protein–protein interaction (PPI) network was constructed based on the STRING database. We identified 30 important hub genes using cytoHubba (Figure 4B). To understand the potential functions of hub genes, we performed GO and KEGG enrichment analyses for these genes. The GO results show that these genes were mainly involved in “epithelial cell proliferation”, “RNA polymerase II transcription regulator complex”, and “DNA−binding transcription factor binding”, etc. (Figure 4C). According to the KEGG enrichment analysis results (Figure 4D), the JAK/STAT pathway, associated with angiogenesis, was enriched. The hub genes associated with this pathway include CCND1, CCND2, STAT2, BCL2, STAT3, and EP300 (Figure S1). A previous study confirmed that the JAK/STAT pathway plays a vital role in angiogenesis, especially the JAK/STAT3 pathway, which plays a key role in angiogenesis [4,25]. Interestingly, VEGFA is a downstream target gene of JAK/STAT3 [26]. Therefore, we hypothesize that CRC-EVs promote angiogenesis by regulating VEGFA by the JAK/STAT3 pathway.

### 3.5. The Promoting Effect of CRC-EVs on HUVECs Was Attenuated by Inhibiting the JAK/STAT3 Signaling Pathway

**Figure 5:**
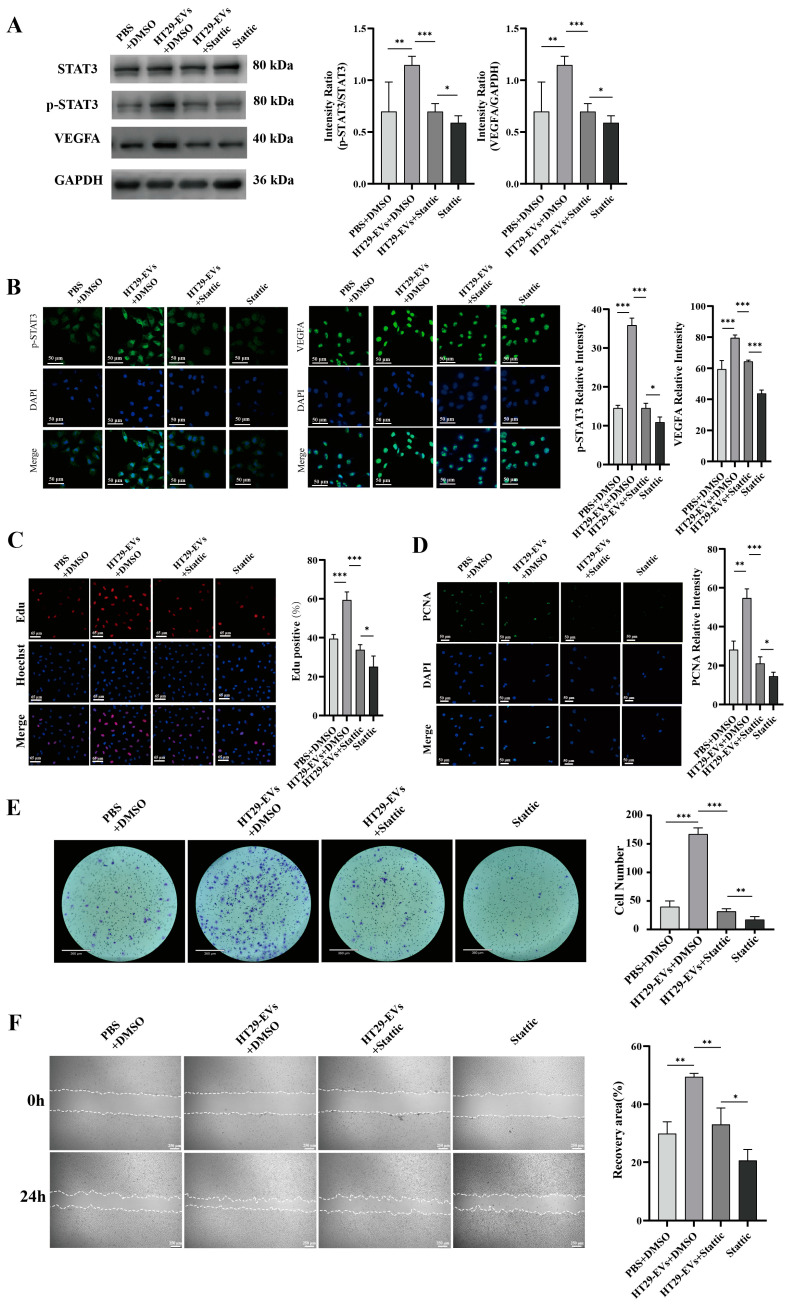

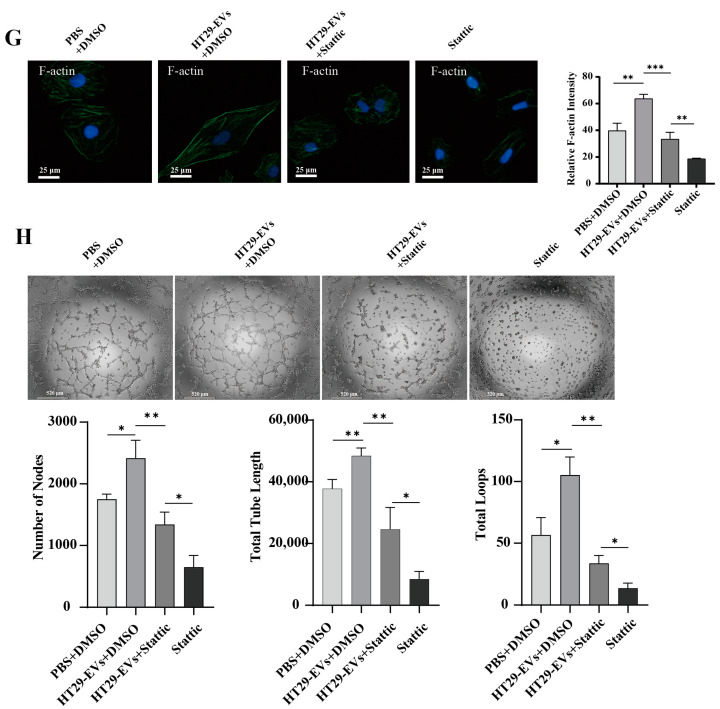
Stattic inhibits the pro-angiogenic effect of HT29-EVs. (**A**) Western blotting analysis of the protein expressions of STAT3, p-STAT3, and VEGFA in HUVECs (Figure S13). (**B**) Immunofluorescence analysis of p-STAT3 and VEGFA protein expression in HUVECs (scale bar = 50 μm). (**C**) The EdU assay was carried out to measure HUVECs proliferation (scale bar = 65 μm). (**D**) Immunofluorescence analysis of PCNA protein expression in HUVECs and fluorescence intensity statistics (scale bar = 25 μm). (**E**) The transwell assay was performed to detect HUVECs migration. (**F**) The scratch wound healing assay was performed to detect HUVECs migration (scale bar = 250 μm). (**G**) The FITC-phalloidin assay was performed to detect F-actin protein expression in HUVECs (F-actin (green) and nuclei (DAPI, blue)). (**H**) The tube formation assay was used to detect the angiogenesis ability of HUVECs (scale bar = 520 μm) and quantitative analysis of nodes, total tube length, and total loops. Data were analyzed by *t*-test, * *p* < 0.05, ** *p* < 0.01, *** *p* < 0.001.

To verify whether CRC-EVs regulate the JAK/STAT3 pathway in HUVECs, we found through Western blotting analysis that the addition of HT29-EVs promoted the expression of p-STAT3 protein, as well as its downstream gene VEGFA (Figure 5A). Additionally, through immunofluorescence, we also found that EVs promoted the expression of p-STAT3 in HUVECs, and the expression of VEGFA was also increased (Figure 5B). Furthermore, EVs derived from CRC cells SW480 and HCT116 (SW480-EVs and HCT116-EVs) also led to increased expression of p-STAT3 and VEGFA (Figure S2).

Further studies showed that by adding a STAT3 inhibitor to suppress the JAK/STAT3 pathway, the expression of p-STAT3 protein was inhibited (Figure 5A,B and Figure S2). Additionally, after adding Stattic to inhibit the JAK/STAT3 pathway, we observed the inhibition of the angiogenic effects of CRC-EVs on HUVECs, including proliferation (Figure 5C,D and Figures S3 and S4), migration (Figure 5E–G and Figures S5–S7), and tube formation (Figure 5H and Figure S8). These results suggest that inhibiting the JAK/STAT3 signaling pathway suppresses the expression of downstream gene VEGFA, thereby inhibiting the angiogenic effects of CRC-EVs.

## 4. Discussion

EVs have been identified as important signaling mediators that regulate the TME [27]. The role of EVs in regulating tumor angiogenesis within the TME is also gradually becoming apparent. For instance, EVs derived from cancer-associated fibroblasts have been shown to promote angiogenesis in colorectal adenocarcinoma cells [28]. Similarly, EVs derived from perivascular cells within tumors also contribute to angiogenesis [29]. Currently, an increasing number of studies have shown that EVs derived from tumor cells play a role in angiogenesis, such as in ovarian cancer [17] and bladder cancer [30]. However, as colorectal cancer is the second most deadly cancer, the role and mechanisms of its EVs in angiogenesis require further investigation. Our research indicates that EVs from HT-29 cells can promote angiogenesis in HT-29 colorectal cancer. In vitro experiments have demonstrated that HT29-EVs can be uptake by HUVECs and promote their proliferation, migration, and tube formation. Our further investigation revealed that CRC-EVs upregulate VEGFA by modulating the JAK/STAT3 pathway, thus promoting the angiogenesis of colorectal cancer tumors.

Angiogenesis plays a crucial role in tumor progression, as tumors rely on the continuous growth of newly formed blood vessels to provide essential nutrients and oxygen [31]. In in vivo experiments, we found that HT29-EVs promote an increase in blood vessels in HT-29 colorectal cancer, leading to increased tumor growth, possibly due to the enhanced supply of oxygen and nutrients from the increased blood vessels. Previous studies have shown that angiogenesis requires the activation of the primary effector cells, specifically endothelial cells, including their migration and tube formation [32]. Our study also revealed that EVs derived from CRC cells promote the proliferation, migration, and tube formation of HUVECs.

VEGFA (Vascular endothelial growth factor-A) is a member of the VEGF family and a key mediator of angiogenesis in cancer [33]. The VEGF family is involved in the formation of new vessels stimulated by hypoxia but also by upregulated factors like cytokines, hormones such as progesterone and testosterone, and transcription factors [34]. Our research has found that HT29-EVs lead to increased expression of VEGFA in HUVECs. Through IHC, we observed that tumors treated with HT29-EVs exhibited increased blood vessel density and higher expression of VEGFA. Previous studies have indicated that tumor-derived EVs regulate tumor angiogenesis through multiple mechanisms. EVs may directly promote endothelial cell angiogenesis by transferring VEGFA [35]. Alternatively, they might regulate target cells through bioactive molecules in EVs, leading to increased intracellular expression of VEGFA and thus enhancing endothelial cell angiogenesis [36]. However, proteomic data from HT29-EVs indicate that VEGFA protein is not present in HT29-EVs [37].

Emerging studies indicate that EVs exert their biological effects by transferring their specific bioactive molecules, such as proteins and miRNAs, especially miRNAs. Many different miRNAs are components of EVs, playing important roles in tumor angiogenesis by regulating various biological processes. For example, in pancreatic cancer, exosomal miR-30b-5p promotes tumor angiogenesis by inhibiting GJA1 [38], while miR-27a promotes angiogenesis by targeting BTG2 [39]. Therefore, to investigate potential mechanisms, we performed differential expression analysis of miRNAs in HT-EVs and FHC-EVs from the GSE40247 dataset. These differential expression miRNAs play crucial roles in the process of cancer development and serve as cancer biomarkers. In order to explore the specific mechanisms by which these differential expression miRNAs regulate angiogenesis, we conducted PPI and pathway enrichment analyses on the top eight DEMs target genes. The results indicate that signaling pathways such as AGE-RAGE, FoxO, and JAK/STAT are enriched. The AGE-RAGE pathway induces angiogenesis through NF-kB [40]. Evidence suggests that FOXOs regulate angiogenesis as both pro- and anti-angiogenic factors [41]. Further studies have indicated that the JAK/STAT signaling pathway regulates tumor angiogenesis [42,43]. Moreover, certain miRNAs that are differentially expressed have been identified as regulators of the JAK/STAT3 signaling pathway. Previous studies have indicated that luciferase reporter assays demonstrate that miR-574-3p directly binds to the 3′ UTR of EP300 [44]. EP300 is a component of the JAK/STAT3 signaling pathway. MiR-296-5p directly targets STAT3 to suppress STAT3 expression [45].

Previous studies have shown that the JAK/STAT pathway plays an important role in angiogenesis [4]. Signal transducer and activator of transcription (STAT) proteins are a cytoplasmic transcription factor family, and among them, STAT3 plays a key role in angiogenesis and is highly activated in most cancers [25]. Typically, STAT3 activation is induced by phosphorylation on a critical tyrosine residue (Tyr 705) that triggers STAT3 dimerization thanks to reciprocal phosphotyrosine SH2 domain interactions [46]. The dimer then translocates into the nucleus, binds to DNA, and exerts its function as a transcription factor [47]. Research has increasingly demonstrated that JAK/STAT3 participates in tumor angiogenesis, particularly in the regulation of VEGFA. VEGFA is a downstream target gene of JAK/STAT3 [26]. Liang et al. discovered that when autophagy was induced by rapamycin, the JAK2/STAT3 pathway was activated and VEGFA was elevated, which was attenuated after deactivating STAT3 by S3I-201 [26]. Zhang et al. discovered that CXCR4 induced VEGF production and JAK2/STAT3 activation and enhanced STAT3 binding to the VEGF promoter, thereby increasing the expression of VEGFA [48]. Therefore, we hypothesize that the angiogenesis mediated by HT29-EVs may be regulated by enhancing VEGFA expression via the JAK/STAT3 pathway.

Stattic, a small-molecule inhibitor of STAT3, inhibits the binding of a physiologically relevant tyrosine-phosphorylated peptide motif to the STAT3 SH2 domain and inhibits dimerization and DNA binding of STAT3 [49], preventing STAT3 from entering the nucleus and exerting its function as a transcription factor, subsequently suppressing the expression of downstream genes. The Western blotting results indicate that HT29-EVs promote the phosphorylation of STAT3 without affecting the expression of STAT3, indicating that HT29-EVs activate the activity of STAT3 and promote the expression of downstream VEGFA. At the same time, we observed that Stattic can significantly reduce the proangiogenic effects of HT29-EVs on HUVECs, including proliferation, migration, and tube formation. This phenomenon indicates that adding Stattic inhibits the JAK/STAT3 signaling pathway, consequently restraining the proangiogenic effects of HT29-EVs on VEGFA.

However, Previous studies have found that Stattic can inhibit the growth of tumors in vivo [50,51]. Our research also suggests a potential mechanism for the inhibition of tumors by Stattic, which may be due to the inhibition of tumor angiogenesis, leading to the suppression of tumor growth.

Our study demonstrates that EVs derived from CRC cells promote tumor angiogenesis by regulating the JAK/STAT3/VEGFA pathway in endothelial cells. In research on epithelial ovarian cancer, EVs promote endothelial cell angiogenesis by activating the JAK/STAT3 signaling pathways [52]. This suggests that there may be more tumor-derived EVs that regulate and promote angiogenesis through the JAK/STAT3 pathway. Future studies may explore how different types of tumor-derived EVs vary in their ability to modulate the JAK/STAT3 pathway and promote angiogenesis. Additionally, research could focus on the potential for targeting EV-mediated signaling in combination with existing treatments to improve therapeutic outcomes in cancer.

## 5. Conclusions

In conclusion, we demonstrated that EVs derived from HT-29 cells enhanced in vivo tumor growth and angiogenesis in CRC by promoting endothelial cell angiogenesis. Bioinformatics analysis and experimental validation revealed that HT29-EVs promote angiogenesis via regulating the JAK/STAT3 pathway to upregulate VEGFA. This study may reveal a new mechanism by which CRC cells induce angiogenesis. Such a mechanism may be used as a target for antiangiogenic therapy.

## Data Availability

The data presented in this study are available on request from the corresponding author.

## Supplementary Materials

The following supporting information can be downloaded at: https://www.mdpi.com/article/10.3390/biology13110873/s1, Figure S1. Cnet plot of enriched KEGG pathways. Figure S2: Western blot analysis of STAT3, p-STAT3, and VEGFA protein expression in HUVECs after different treatments. Figure S3: The EdU assay was carried out to measure HUVECs proliferation. Figure S4: Immunofluorescence analysis of PCNA protein expression in HUVECs and fluorescence intensity statistics. Figure S5: The transwell assay was performed to detect HUVECs migration. Figure S6: The scratch wound healing assay was performed to detect HUVECs migration (scale bar = 560 μm). Figure S7: The FITC-phalloidin assay was performed to detect F-actin protein expression in HUVECs. Figure S8: The tube formation assay was used to detect the angiogenesis ability of HUVECs. Figure S9: Nanoparticle tracking analysis of the size distribution of the extracellular vesicles derived from SW480 and HCT116. Figure S10: Transmission electron microscopy image of the extracellular vesicles derived from SW480 and HCT116. Figure S11: Western blotting analysis of the positive (CD63 and TSG101) and negative (calnexin) markers extracellular vesicles derived from SW480 and HCT116. Figure S12: Uncropped western blot images that correspond to Figure 3H. Figure S13: Uncropped western blot images that correspond to Figure 5A.
