# Supplementary material for: Colorectal Cancer Cell-Derived Extracellular Vesicles Promote Angiogenesis Through JAK/STAT3/VEGFA Signaling
Source: Biology (Basel). 2024 Oct 27;13(11):873. doi: 10.3390/biology13110873 (PMC11591796; doi:10.3390/biology13110873)
Supplement: Supplementary file 1 [file biology-13-00873-s001.zip › biology-3181690-supplementary.pdf]

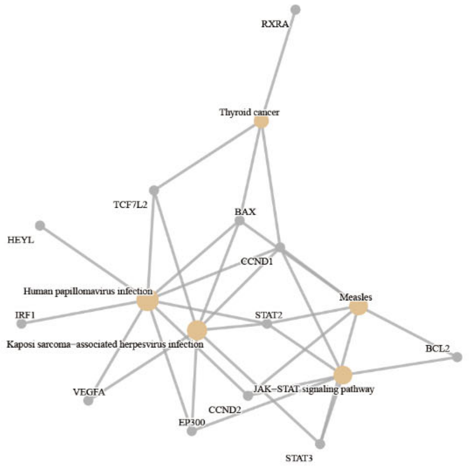

Figure S1. Cnet plot of enriched KEGG pathways.

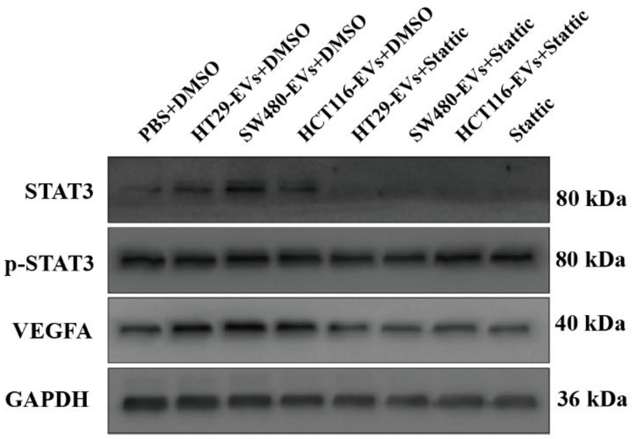

Figure S2. Western blot analysis of STAT3, p-STAT3, and VEGFA protein expression in HUVECs after different treatments.

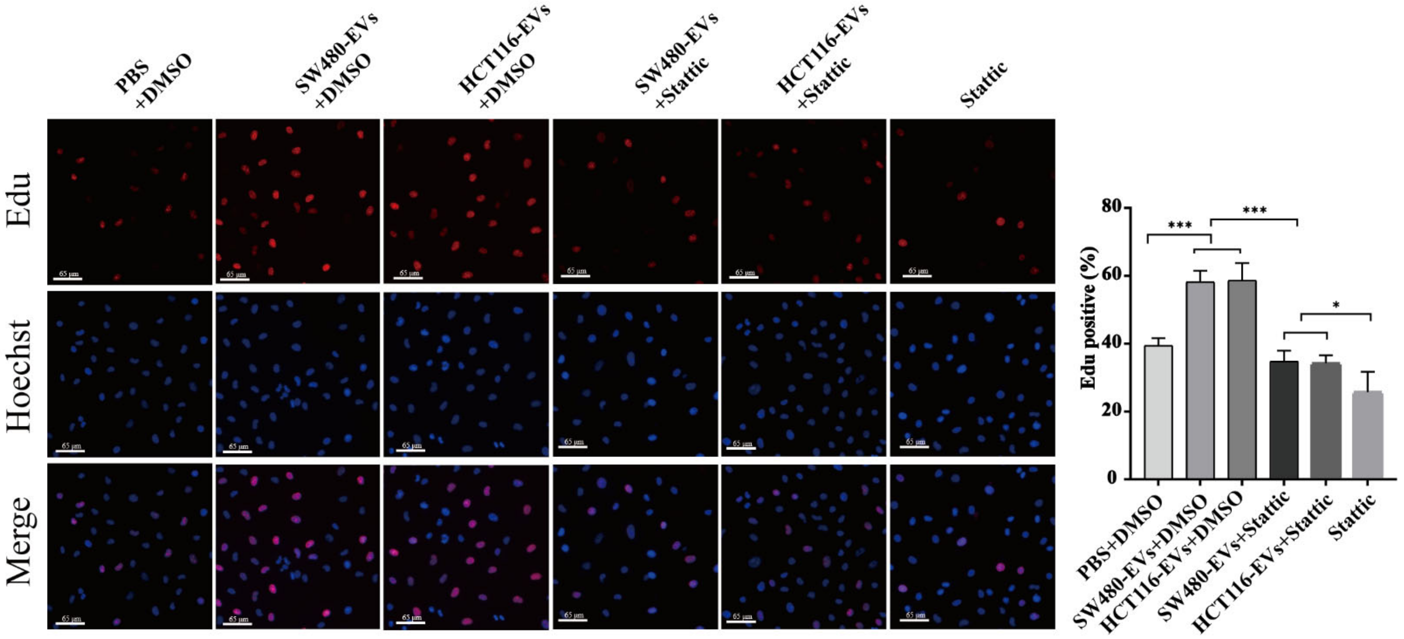

Figure S3. The EdU assay was carried out to measure HUVECs proliferation (scale bar = 65 μm).

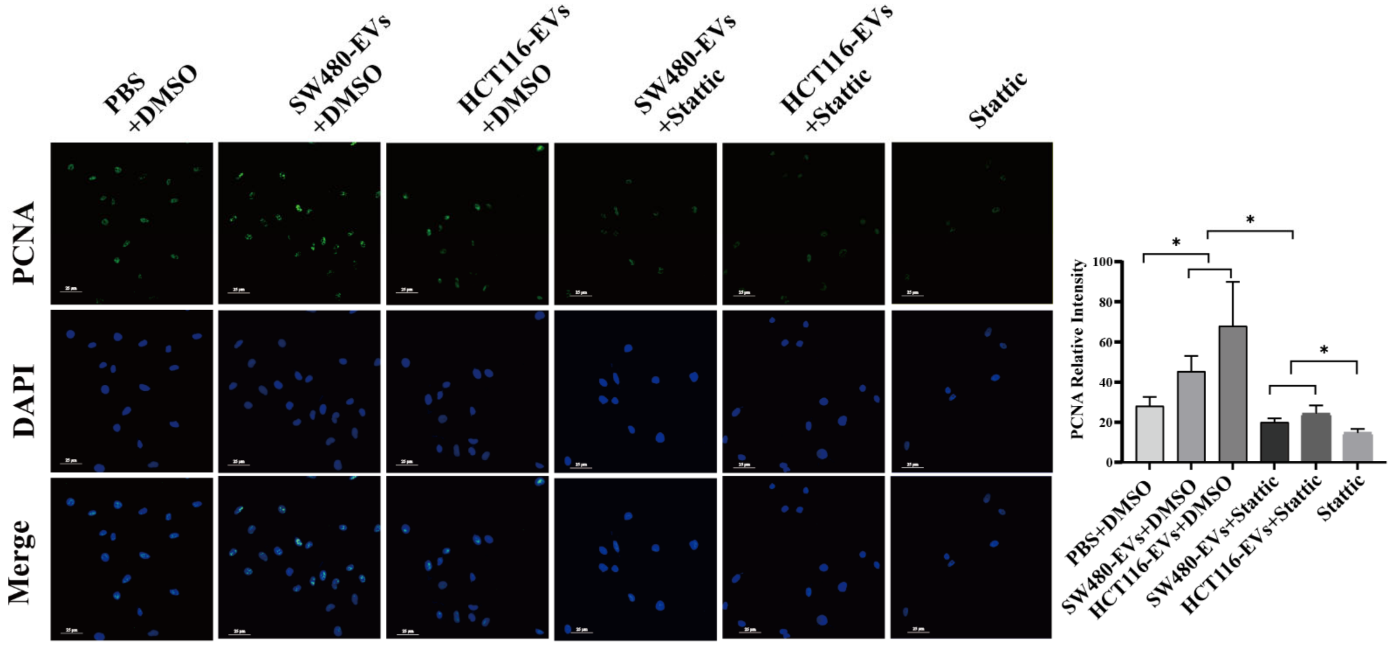

Figure S4. Immunofluorescence analysis of PCNA protein expression in HUVECs and fluorescence intensity statistics (scale bar = 25 μm).

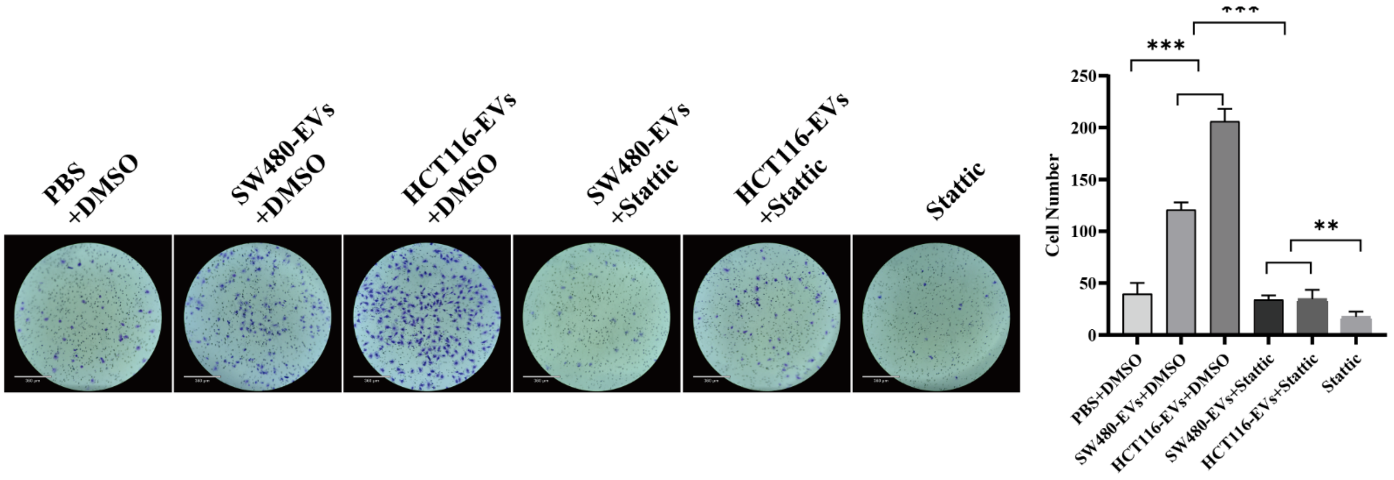

Figure S5. The transwell assay was performed to detect HUVECs migration.

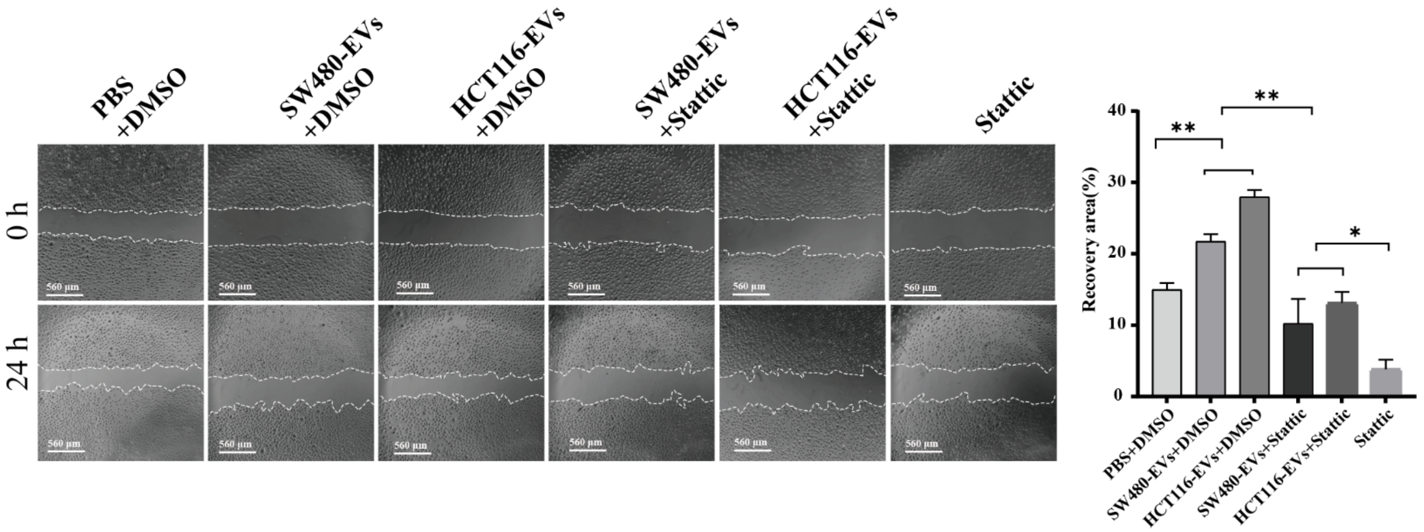

Figure S6. The scratch wound healing assay was performed to detect HUVECs migration (scale bar = 560 μm).

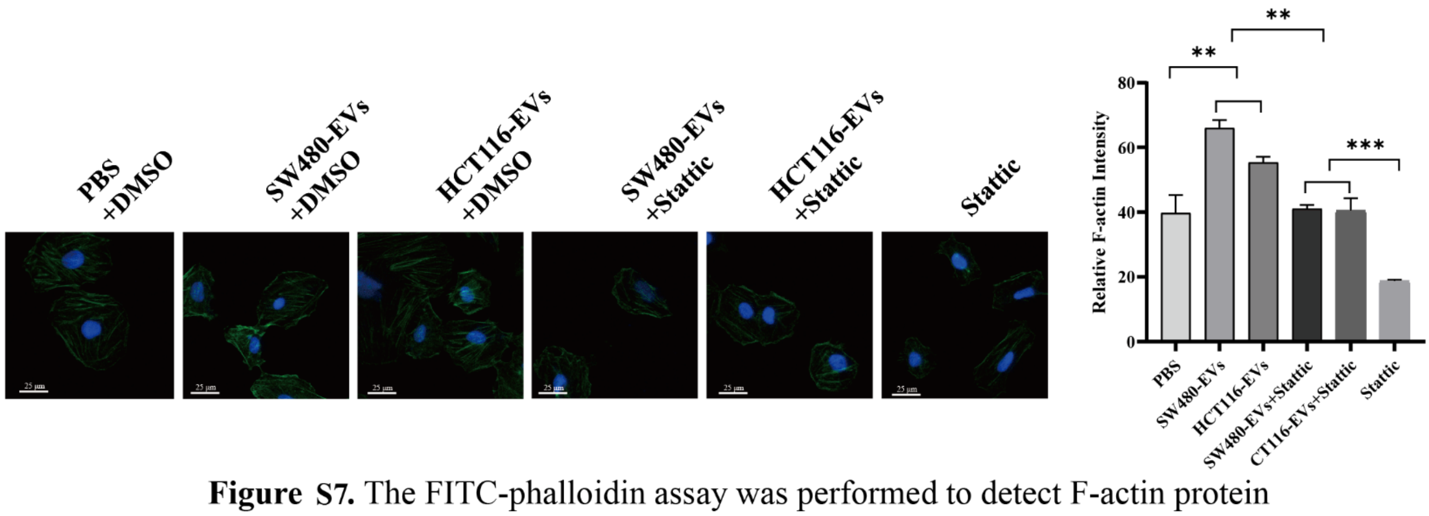

Figure S7. The FITC-phalloidin assay was performed to detect F-actin protein expression in HUVECs (scale bar = 25 μm).

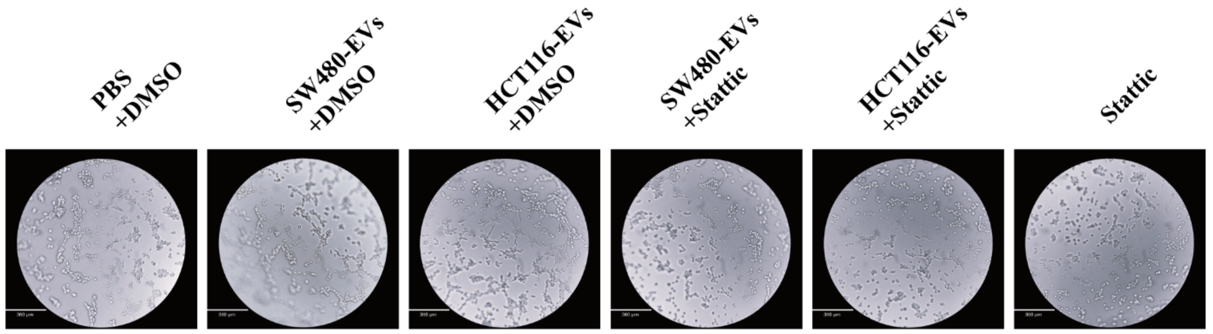

Figure S8. The tube formation assay was used to detect the angiogenesis ability of HUVECs.

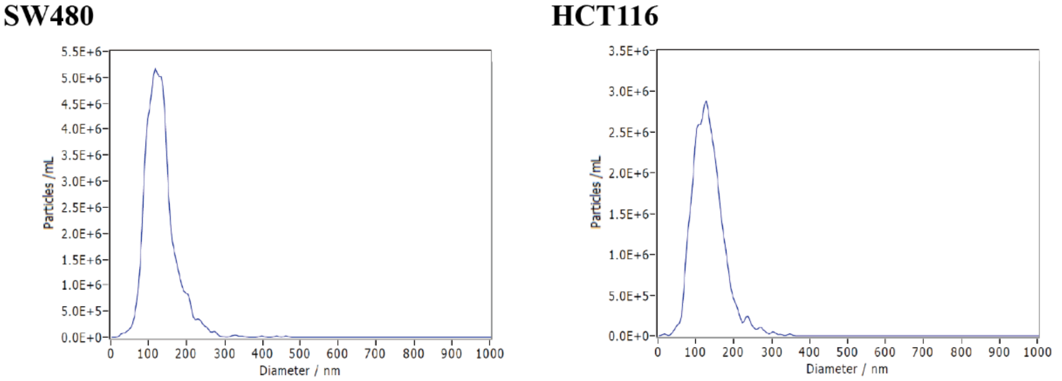

Figure S9. Nanoparticle tracking analysis of the size distribution of the extracellular vesicles derived from SW480 and HCT116.

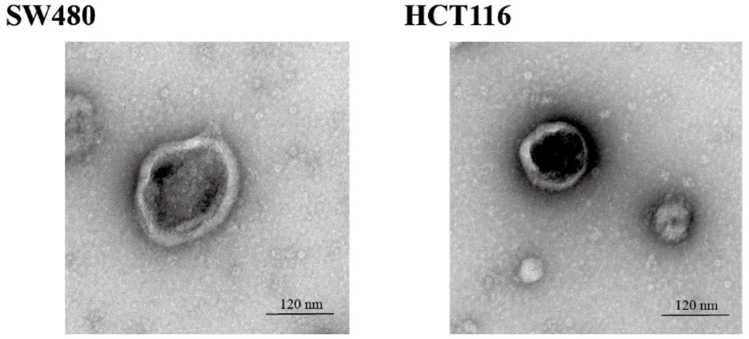

Figure S10. Transmission electron microscopy image of the extracellular vesicles derived from SW480 and HCT116.

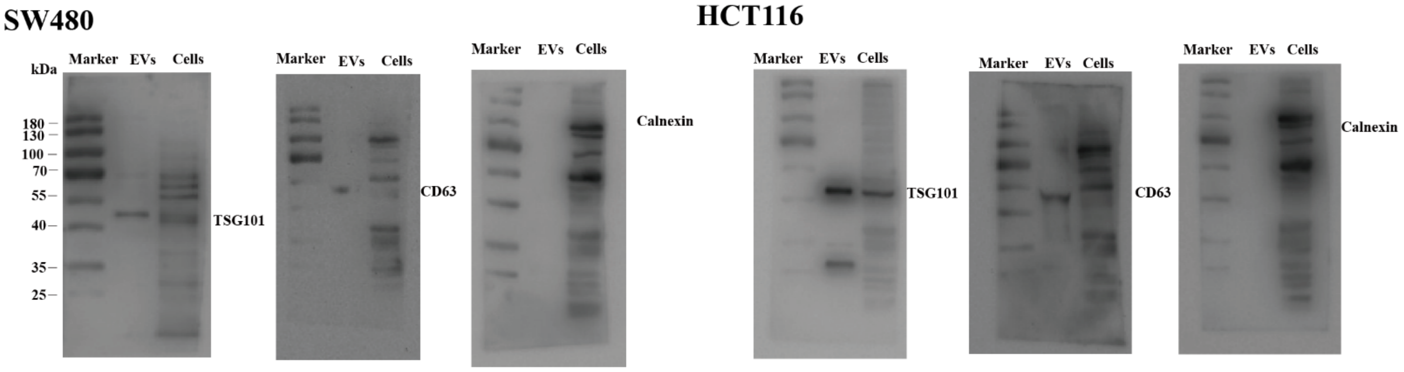

Figure S11. Western blotting analysis of the positive (CD63 and TSG101) and negative (calnexin) markers extracellular vesicles derived from SW480 and HCT116.

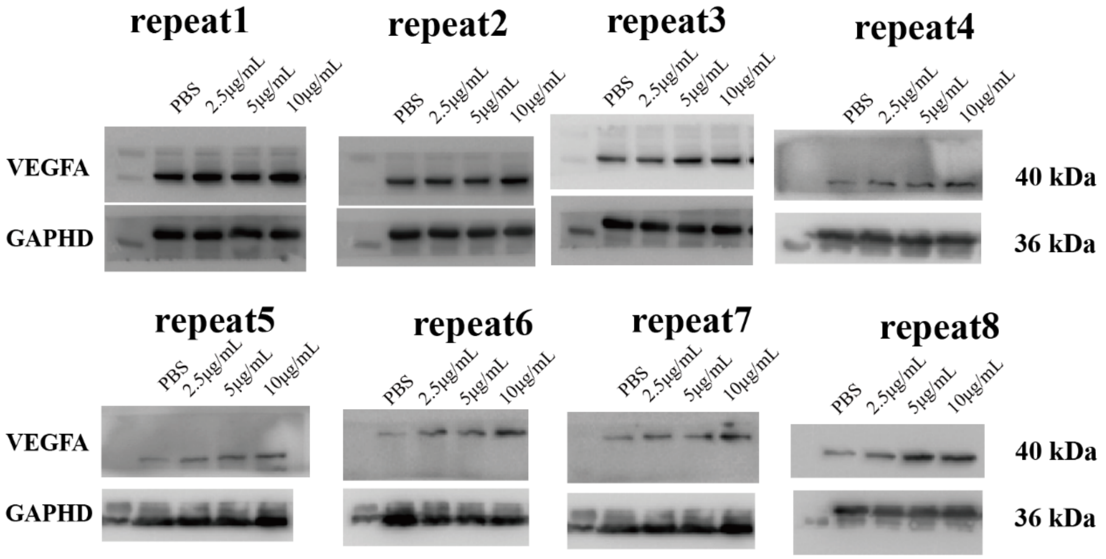

Figure S12. Uncropped western blot images that correspond to Figure 3H.

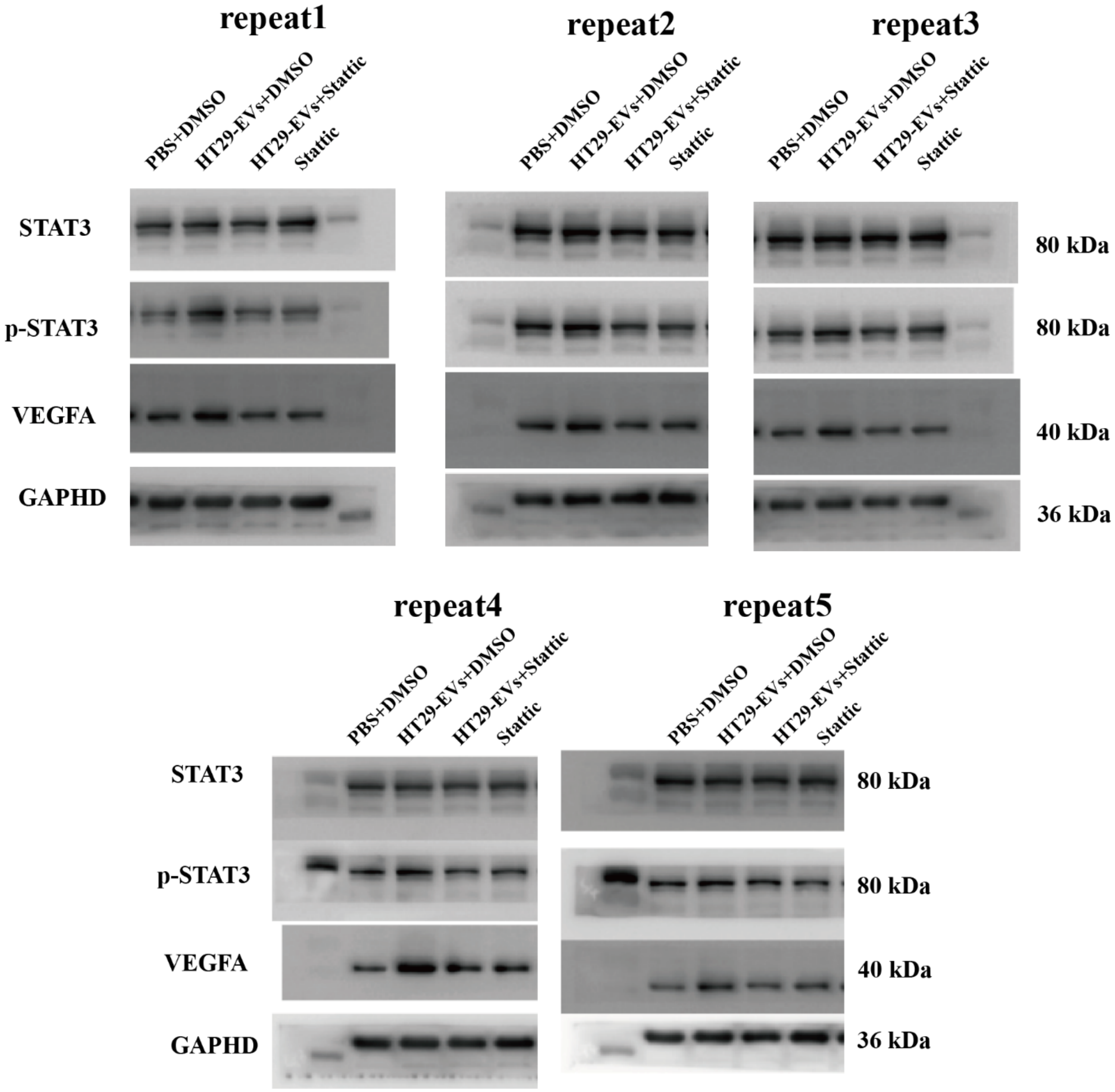

Figure S13. Uncropped western blot images that correspond to Figure 5A.
